# The Social Burden of Resilience: A Historical Perspective

**DOI:** 10.1007/s10745-018-0002-2

**Published:** 2018-06-03

**Authors:** Adam Izdebski, Lee Mordechai, Sam White

**Affiliations:** 1 Institute of History, Jagiellonian University in Krakow, ul, Golebia 13, 31-007 Krakow, Poland; 2 Max Planck Institute for the Science of Human History, Kahlaische Str. 10, Jena, 07745 Germany; 3 Medieval Institute, University of Notre Dame, Notre Dame, IN USA; 4 Department of History, Ohio State University, Columbus, OH USA

**Keywords:** Resilience, Social differentiation, Roman Empire, Byzantium, Ottoman empire

## Abstract

We examine the social burden associated with resilience to environmental shocks in pre-modern societies. We argue that analyses of state-level interventions to mitigate the consequences of catastrophic events tend to isolate these measures from their larger social contexts and thereby overlook the uneven distribution of their burden across different groups. We use three cases of pre-modern societies in the northeastern Mediterranean - the sixth century Roman Empire, the tenth century Byzantine Empire, and the sixteenth century Ottoman Empire. We demonstrate how the adaptive processes that reinforced resilience at the state level incurred different burdens for those at lower levels of the social hierarchy. We found that some groups sustained losses while others gained unexpected benefits in the context of temporary systemic instability. We also found that although elites enjoyed enhanced buffers against the adverse effects in comparison with non-elites, this did not consistently guarantee them a better outcome. We conclude that the differentiated burden of resilience could in some cases entrench existing political or economic configurations, and in other cases, overturn them. Our case studies indirectly address the pressing issue of environmental justice.

## Introduction

The popularity of resilience in academic literature and policy-making has increased over the past few decades (Folke 2006). Resilience, in the sense of bouncing back after an unexpected shock, was originally used to conceptualize natural systems. The term was soon introduced to the social systems and even the humanities (e.g., for archaeology Redman and Kinzig 2003; Redman 2005). As its use expanded, however, some have argued against its usefulness (Joseph 2013; Olsson *et al*. 2015).

Social science research usually presents resilience as an attractive attribute or goal of economic and political systems. Analyses of resilience may therefore influence decision-makers seeking to achieve resilience for a business, agency, or entire government. In these contexts, the pursuit of resilience by one organization may be isolated from its effects on sub-, supra-, and parallel systems. How the resilience of one component of a society affects other groups and institutions within the same society is therefore less frequently discussed (Olsson *et al*. 2015).

Nevertheless, resilience often incurs costs, which are often externalized and unevenly distributed across different social classes and environments. Although theoretically possible, cases in which resilience benefits everyone and everything alike are rare. Policy-makers pursuing resilience must face questions raised by Cote and Nightingale (2012) about resilience “of what” and in particular “for who;” these questions may in turn raise ethical dilemmas regarding the maintenance of power structures and vested interests. For example, the scholarly discourse of resilience as a concept had incorporated neo-liberal ideals such as an emphasis on individual adaptability from an early point (e.g., Holling 2001; for neo-liberalism see Harvey 2005). Subsequent scholars have critiqued the concept and in particular its use in policy literature, asserting that the discourse surrounding resilience perpetuates neo-liberal views of governance (Joseph 2013; for similar developments in climate impact studies, see Hulme 2011).

The three case studies below examine complex societies in the northeastern Mediterranean (the Roman, Byzantine, and Ottoman empires) and the ways in which they spread the social burden of resilience across their different constituent groups. Each occupied a similar geographical region where they represented the dominant political, economic and cultural force. They proved to be sustainable and adaptable socio-political entities that survived and thrived for centuries. Despite changes of names, dynasties and religions, there were a number of key structural continuities between them, especially in the case of the Roman and Byzantine empires.

Our research concerns the socio-cultural burden of resilience in these three complex pre-modern societies. Each encountered socio-environmental challenges in the form of natural and social disasters, adverse climate and pathogenic disease, which interacted with the system’s vulnerabilities. In each case, the state appears to be a resilient social structure that was able to mitigate the socio-environmental effects and adapt to a new reality, maintaining its continuity over time. However, a closer investigation of the social elements that constitute the larger system (whether socio-economic groups such as elites or peasantries, or institutions such as aspects of the state’s operations, or the church, for example), reveals that the costs—and benefits—of its resilience were not divided equally. Rather, in each case the benefits and burdens of resilience were distributed across society unevenly. Resilience (or vulnerability) in one sector of a society directly impacts its other sectors in different ways.

Our research corresponds to two of the key questions of historical ecology raised by Armstrong *et al*. (2017): (1) How did past societies respond to sudden environmental shocks? (2) What factors have made some communities more adaptable to environmental stress than others? It also responds to Adamson *et al*. (2018), who encouraged more nuanced uses of the past in the study of how societies adapt to climate change.

Since the environment interacts with human societies rather than determines their fate, environmental effects are never uniform for all parts of a society (e.g., Adger 2001; Houston 2008; Sandoval *et al*. 2014). Each subgroup of a complex society has its own set of vulnerabilities and thus will be affected in a different manner as a result of its particular interaction with the environment. For example, we would expect that a drought would have differential effects on farmers living in a village compared to bureaucrats living in the nearby city. Even highly destructive events are socially patterned: the isolated, weak, and less wealthy consistently fare worse (Matthewman 2015: 20), while certain groups can benefit even when society as a whole suffers (Campbell 2016). Cutter *et al*. (2003) aggregated about 20 general factors that can affect the social vulnerability of individuals or groups. Using modern data, they found that the most important of these were personal wealth, age, density of the built environment, single-sector economic dependence, and housing stock and tenancy. Together, these five factors explain about 50% of the variability in social vulnerability at the US county level. Except for age, which remains unknown for practically all pre-modern populations at the group level, the other four factors play a major role in the pre-modern case studies below.

In our analysis we follow Steadman and Ross (2017) and Tainter (1988) and define complex societies as those that possessed a common set of institutions beyond kinship bonds that guided their political, social, economic, and religious organization. Social complexity is thus an abstract, continuous variable, which is typically correlated with increased population, inequality, and heterogeneous occupational specialization.

## Case Studies from the Premodern Eastern Mediterranean

### The Sixth Century: Disasters and Civic Response in the Roman Empire

The Levant was one of the richest regions of the Roman Empire. The interregional trade routes that passed through it mixed people, goods, and ideas. Speckled with large cities, it was a cultural and religious center, home to a wealthy, educated, and Romanized population. This idyllic picture, however, was shattered over the sixth century when frequent earthquakes, multiple waves of bubonic plague, and devastating enemy attacks strained established political and economic structures.

**Fig. 1:**
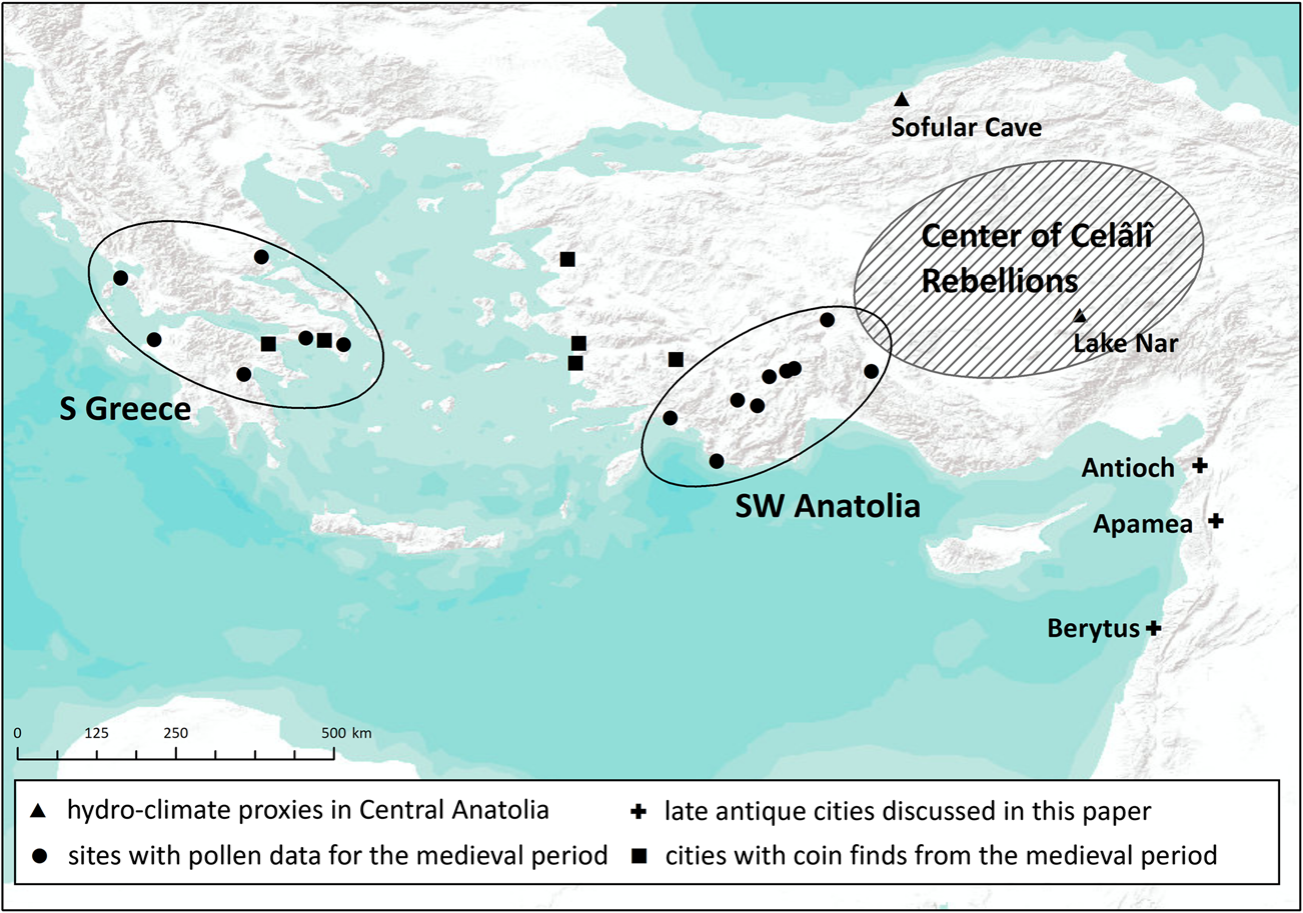
Sites and areas discussed

Relative to modernity, very little evidence survives for late antiquity and researchers are dependent chiefly on the results of archaeological excavations and a few historical texts that describe politics and war far more frequently than social, cultural, and environmental developments. Within the region, we know most about the central city of Antioch (Antakya or Hatay in southeastern Turkey), which experienced more than 36 disasters over the century (Mordechai 2017). Despite the paucity of direct evidence for other settlements in the region, they must have experienced many of the same events as Antioch. To simplify the analysis, we examine this ancient metropolis and two other cities: Apamea (Afamiya, Syria), 90 km south of Antioch, and Berytus (Beirut, Lebanon), another 190 km southwest. Antioch and Apamea were capitals of their provinces while Berytus was a major cultural center (Fig. 1).

Enough independent types of evidence survive to convey the extent of disruption. Seismic activity in the region increased fourfold over the sixth century (Khair *et al*. 2000). Antioch itself experienced several major earthquakes over the period (Guidoboni 1994; Ambraseys 2009). Contemporaries described these events, which would affect other settlements as well, in stark terms such as “almost the whole of Antioch collapsed in ruins” (Chronicle of 724, 143 in Guidoboni 1994: 346) and “an unquantifiable multitude [of people] was caught” in the destruction of Antioch (Evagrios Scholasticus, trans. Whitby 2000: 6.8). Antioch further suffered, among others, from a major fire (525), a Persian sack (540) after which the city was burned and its residents forcibly resettled in Persia, and at least four “waves” of the Justinianic plague (beginning in the early 540s). Only a few scattered anecdotes describe the fate of Apamea and Berytus. The former was plundered by the Persians in 540, and again in 573, when a Persian general looted the city, captured its inhabitants and burned it (Foss 1997: 205-229). The earthquake of 551 caused substantive damage to Berytus (Darawcheh *et al*. 2000; Hall 2004).

From the empire’s perspective, this series of disasters endangered its hold on some of its wealthiest regions. Furthermore, imperial obligations elsewhere limited the resources at the government’s disposal. Yet despite such challenges, the state maintained its control over Syria throughout the century. Its strategies included direct investment in the region and outsourcing some of the tasks to locals through coercion. Altogether, the sixth-century imperial system demonstrated outstanding resilience at the state level. A closer examination, however, reveals a more nuanced reality at the city level and even more variability among the social groups within each city.

Although one might expect Antioch to have collapsed after so many disasters, the city survived. While it had not completely recovered by the time of the Persian conquest (see below) there is enough evidence to attest to its continued great size and importance in the late sixth and early seventh centuries (see for example Evagrios Scholasticus (6.8) who claims that 60,000 people died in 588, or John of Ephesus 226 [in Payne Smith 1860]). A number of key factors might explain this surprising outcome.

First, contemporary emperors took a personal interest in the city’s recovery and wellbeing. For this purpose they infused it with cash and implemented large-scale reconstruction projects. Government officials also allocated resources of their own to rebuild and repopulate Antioch after disasters (Procopius *Buildings*, Dewing 1914a: 2.10; Downey 1961: 548-553; Malalas, Jeffreys *et al*. 1986: 421-24, 444, 450, 452, 470-1; Evagrios Scholasticus 6.8). The imperial government further signaled its commitment to maintain Antioch as a major city through a public relations campaign and free bread doles (e.g., the city’s name was changed to Theoupolis, literally “The City of God,” Malalas 443; Evagrios Scholasticus 6.8).

In the wake of the numerous disasters that Antioch experienced during the century, these measures lured more inhabitants into the city. For a modern comparison, the post-destruction rebuilding efforts after the 1908 earthquake in Messina (Sicily), which killed about half of the city’s pre-earthquake population (about 150,000), also offered attractive new opportunities: state-funded jobs, cheap land, and even looting the ruins. As a result, Messina’s population swelled to 118% of its pre-earthquake size in 13 years (Restifo 1995; Parrinello 2012). Similar trends took place in late antique Antioch as people from its hinterland and other cities in the region resettled in it and benefited from parallel economic opportunities. Unfortunately for the residents, the rebuilding process was cut short by the early seventh century Persian conquest, which focused the historical narrative on the city’s decline (e.g., Downey 1961).

Apamea enjoyed far less governmental attention than Antioch: none of the historical sources that refer to it over the sixth century mention any type of imperial support following the disasters that afflicted it. Prokopios’ *Buildings*, a panegyric to emperor Justinian (527-565) for his building activities throughout the empire, includes an entire chapter about Antioch but does not mention Apamea at all (Procopius *Buildings,* Dewing 1914a: 2.10, 5.9.27). Furthermore, few of the dozens of sixth-century inscriptions in the area mention the emperor as a sponsor or in an invocation (Trombley 1997).

Apamea was left to its own devices but was not depopulated or abandoned. The city’s main street and walls were rebuilt - actions attributed to emperor Justinian although there is no concrete evidence for his involvement. Building projects in the area continued into the seventh century but were undertaken by local elites, many of whom did not list any affiliation they may have had with the central government in surviving inscriptions. This alienation could explain why Apamea’s citizens preferred to surrender each time the city was threatened (540, 573, 610/1 and ca. 640). In 540 the citizens *de facto* accepted the Persian king as their ruler (Procopius, *Wars*, Dewing 1914b: 2.11.14), whereas during the Arab conquest (ca. 640) they supposedly greeted the Arab armies with tambourine players and singers (al Baladhuri, *Futuh*: 131). Similar capitulations took place in 573 (John of Ephesus, Payne-Smith 1860: 6.6) and 610/1 (Foss 2003). Archaeological evidence suggests that the city’s elite eventually emigrated to Constantinople, while the new economic opportunities in the city lured people from its surroundings who moved into the houses of the now-absent elite (Balty 1984a). These migrations changed the nature of the city and its society became less stratified. Estimating Apamea’s population is difficult, but coin use in the city appears to have increased until the late eighth-early ninth century, hinting at more economic activity (Balty 1984b; Nègre 1984).

Berytus, “the jewel of Phoenicia” (Agathias, *Histories*, Frendo 1975: 2.15.2), was a smaller city but a cultural and economic center. It was a key point for the silk industry, importing raw material from the east and processing it before shipping it around the Mediterranean. It also housed a renowned law school that “conferred an aura of peculiar prestige and distinction on the place” (Agathias, *Histories*: 2.15.3) and was attended by students from all over the empire. In the 530s and 540s, centralizing policies drew much of the silk trade and several of the law school’s faculty members to the capital Constantinople (Procopius, *Secret History*, Dewing 1914c: 25.13-26; the law school professors went to revise the law, see Hall 2004: 212-213). While both institutions probably survived, they would have been left in a more vulnerable state after losing human and material capital.

According to both literary and archaeological evidence, the earthquake of 551, which coincided with a tsunami and caused a major fire, destroyed parts of the city, including the facilities used for teaching (e.g., Agathias, *Histories:* 2.15.1-4; Saghieh 1996: 40). Estimates put its local magnitude at 7.3-7.8 and its epicenter was probably a few kilometers off the shore of the city (Darawcheh *et al*. 2000; Elias *et al*. 2007). The surviving written sources agree that the emperor sent some funds for relief and reconstruction efforts (Hall 2004: 70-75).

**Fig. 2:**
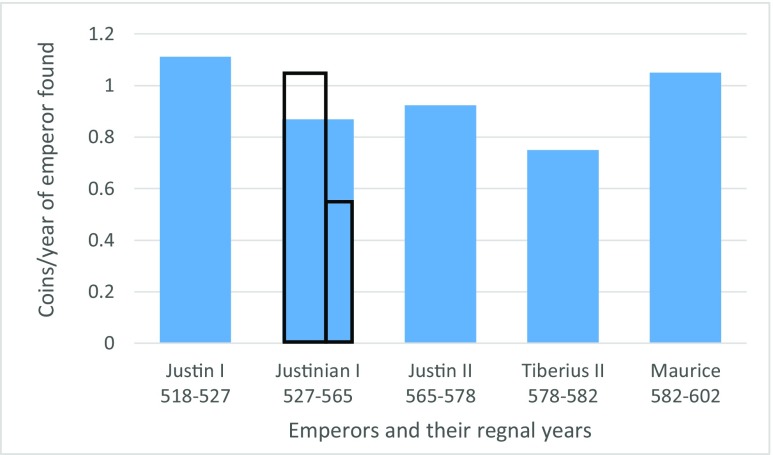
Coins per year in the reigns of emperors between 518 and 602, found in Berytus excavations. The two black outlines represent the ratios before and after the earthquake of 551. The coin data (which does not include hoards) was taken from Butcher 2001-2002; Finkbeiner and Sader 1997; Nurpetlian 2016a; Nurpetlian 2016b

There is no evidence that the law schools or the silk industry ever returned to Berytus, and contemporaries lamented the city’s destruction after the earthquake (Piacenza Pilgrim: 159 in Wilkinson 1977: 79; Iohannes Barbucallus in Paton 1917: 236-237). Although the city disappears from the written sources almost completely after the vague reference to rebuilding, archaeological evidence reveals surprising continuity and even innovation. The ceramic evidence, for example, shows that local workshops continued to produce pottery but changed their fabric and firing methods and introduced different designs. New pottery imports from northern Palestine appear on the site, while there is evidence for the city shipping its local amphorae after a long hiatus (Reynolds 2000:391; Reynolds and Waksman 2007:61). The plentiful glass material found at the site suggests that there were enough raw materials. The number of glass lamps increased dramatically and new forms began appearing after the earthquake (Jennings 2004-5: 134, 185 and *passim*). The published coin material from the city, which can be dated with more precision than the other types of evidence, shows only a brief drop after the earthquake, which could be interpreted as evidence for a short-term decline in monetary activity (Fig. 2).

The central government and its actions had a direct influence on the trajectory of at least some of the groups in Berytus. Its centralizing efforts weakened both the law school and the silk industry in Berytus, which collapsed as a result of the earthquake, incidentally benefiting the Constantinople silk industry and law school. After the earthquake the government also contributed at least partially to the reconstruction of Berytus, facilitating its survival.

In all three cases, therefore, the state’s resilience had significant costs at the city level that were determined by the interaction between the environment, the state, and specific cities. We know most about the central government’s actions: its commitment and support-maintained Antioch as a major center, but its lukewarm treatment of Apamea undermined the loyalty of local elites. The environmental stress in Berytus coincided with the city’s government-induced vulnerabilities, resulting in the collapse of its most famous institutions.

This analysis preserves echoes of the choices individuals had to make within the parameters determined by the interaction between the government and the environment. Villagers around Antioch who lost the markets to which they exported their products, people who lost their homes in earthquakes, or the silk merchants in Berytus were all likely to readjust their lives to fit a new reality. Others, such as the government contractors who provided the free bread for Antioch, those who worked at the law schools in Constantinople, and even the immigrants who took over the mansions in Apamea all benefited from the same realities.

The extent to which the state was aware of these trends or tried to reverse them remains unknown because of the lack of sources. Nonetheless, the elite officials that made up the government must have experienced conflicts of interest between advancing the collective goals of the institution they led and their private gain. Such factors, perhaps just as much as strategic planning, would have determined the nuanced patterns of resilience in sixth century Roman society.

### The Tenth Century: Severe Winter and Social Transformation in Byzantium

The severe winter and consequent great famine of 927 CE in the Byzantine Empire represent a classic case of a major climate-related subsistence crisis that accelerated the pace of social change, creating opportunities for some social groups at the same time as aggravating the situation of others. We know the events in relative detail thanks to contemporary imperial legislation dealing with provincial land ownership for most of the tenth century (927-996). This collection of legal documents contains a number of highly rhetorical passages within key pieces of state legislation, which set out the reasons for their promulgation, providing valuable information on their context (analysed in detail in Kaplan 1992: 414-426; edition of the Greek text: Svoronos 1994; English translation: McGeer 2000). The winter and the subsistence crisis of 927 CE are also described in Byzantine hagiographical and historical accounts (Morris 1976; Telelis 2004: 373). Taken together, these sources provide a fairly good understanding of both the environmental and societal aspects of these events.

**Fig. 3:**
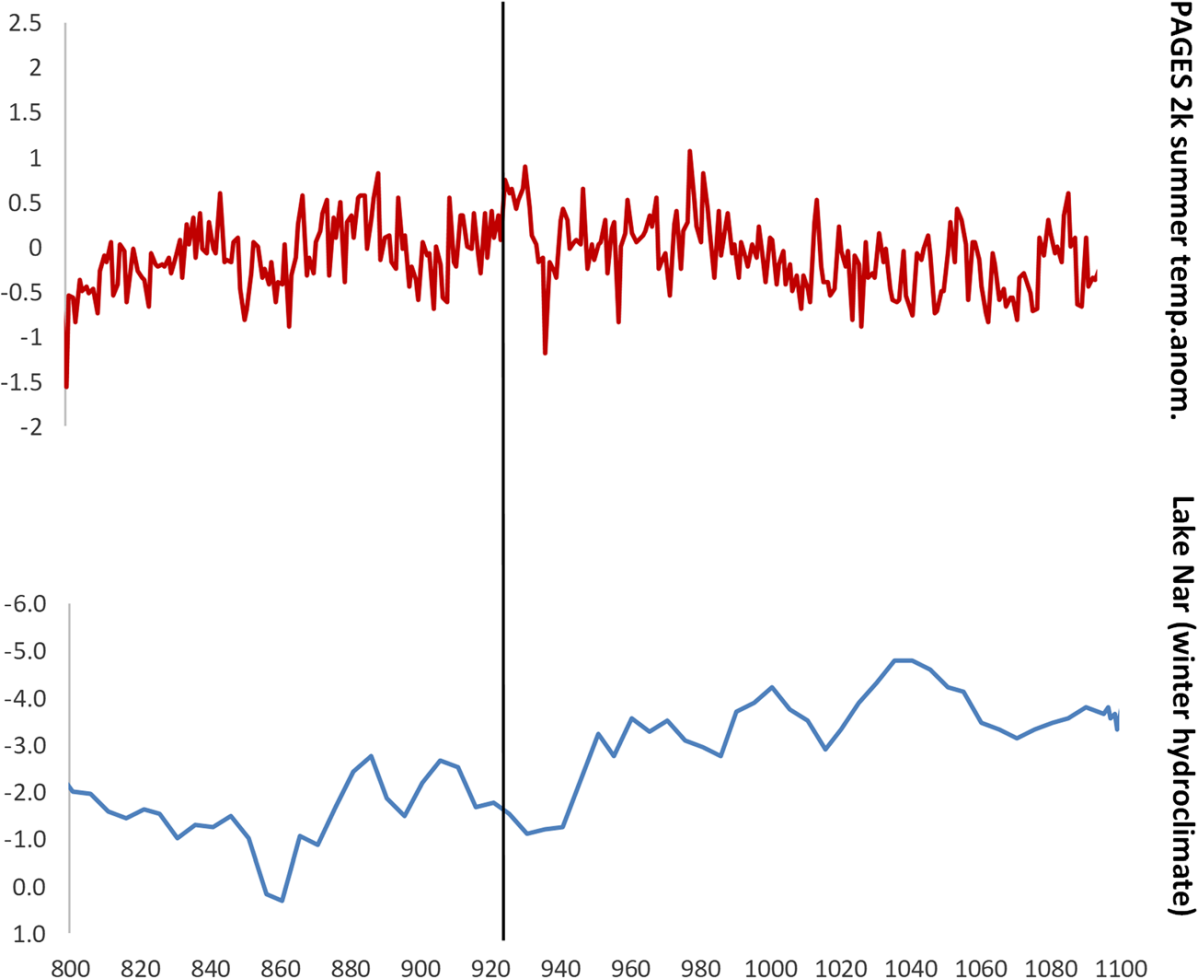
Annually-precise palaeoclimate proxies for the Byzantine Empire. The black line marks the year AD 927. Lake Nar δ18O data: Jones et al. 2006 values are reversed so that the higher values represent wetter winter conditions); summer temperature anomalies are relative to 1961-1990 (in °C): PAGES 2k Consortium 2013 (r correlation value between the summer and annual temperature reconstructions: 0.66)

In the Byzantine cultural memory, the winter of 927-28 stands out as particularly severe, and this period later was remembered as a time of “Great Famine.” Tenth-century sources describe the winter as characterized by an unusual cold spell that lasted for as long as 120 days, starting on Christmas 927 CE. It is unfortunately difficult to translate the reports of the sources into more precise spatial terms: one source tradition is very general and gives no geographical details, while the other one may be coming from Anatolia (Delehaye 1923: 205 (for Anatolia); Wahlgren 2006: 330; other sources in all probability deriving from the ones referenced here in Telelis 2004: 373). Surprisingly, whereas the Byzantine sources unanimously describe the winter as unusually long and cold, no currently available palaeoclimate proxy confirms the occurrence of a strong winter cooling, or at least a potential increase in snowfall, at that time. An annually-precise regional hydroclimate proxy from Central Anatolia, Lake Nar, which reflects winter precipitation, shows no significant variability for the decades of 920s and 930s (Lake Nar chronology is based on varve-counting: Jones *et al*. 2006; similar conclusions can also be reached when looking at a chronologically less precise hydroclimate proxy from north-western Anatolia, the Sofular Cave: Göktürk *et al*. 2011; for snowfall and winter tempratures, see the Kocain cave data, Göktürk  2011). The summer temperature reconstructions for Europe – strongly correlated with the annual temperature, with the *r* value of 0.66 – suggest a steady increase in warmth for the summer months over the 920s and 930s until a major volcanic eruption occurred in 939 CE (PAGES 2k Consortium 2013, correlation value: Table 1; Toohey and Sigl 2017; Oppenheimer *et al*. 2018) (Fig. 3). Moreover, recent palaeoclimate model simulations for the entire Aegean area suggest that throughout the 920s average temperatures remained relatively stable (Xoplaki *et al*. 2016). To conclude, it seems that while the winter of 927 CE in Anatolia and the Southern Balkans (the core territories of the Byzantine Empire at that time) might have been colder and longer than usual at that time, as reported in the written sources, there occurred no major general cooling and some winters during the later tenth century might have actually been much colder, as suggested by the European temperature anomalies (Fig. 3).

**Fig. 4:**
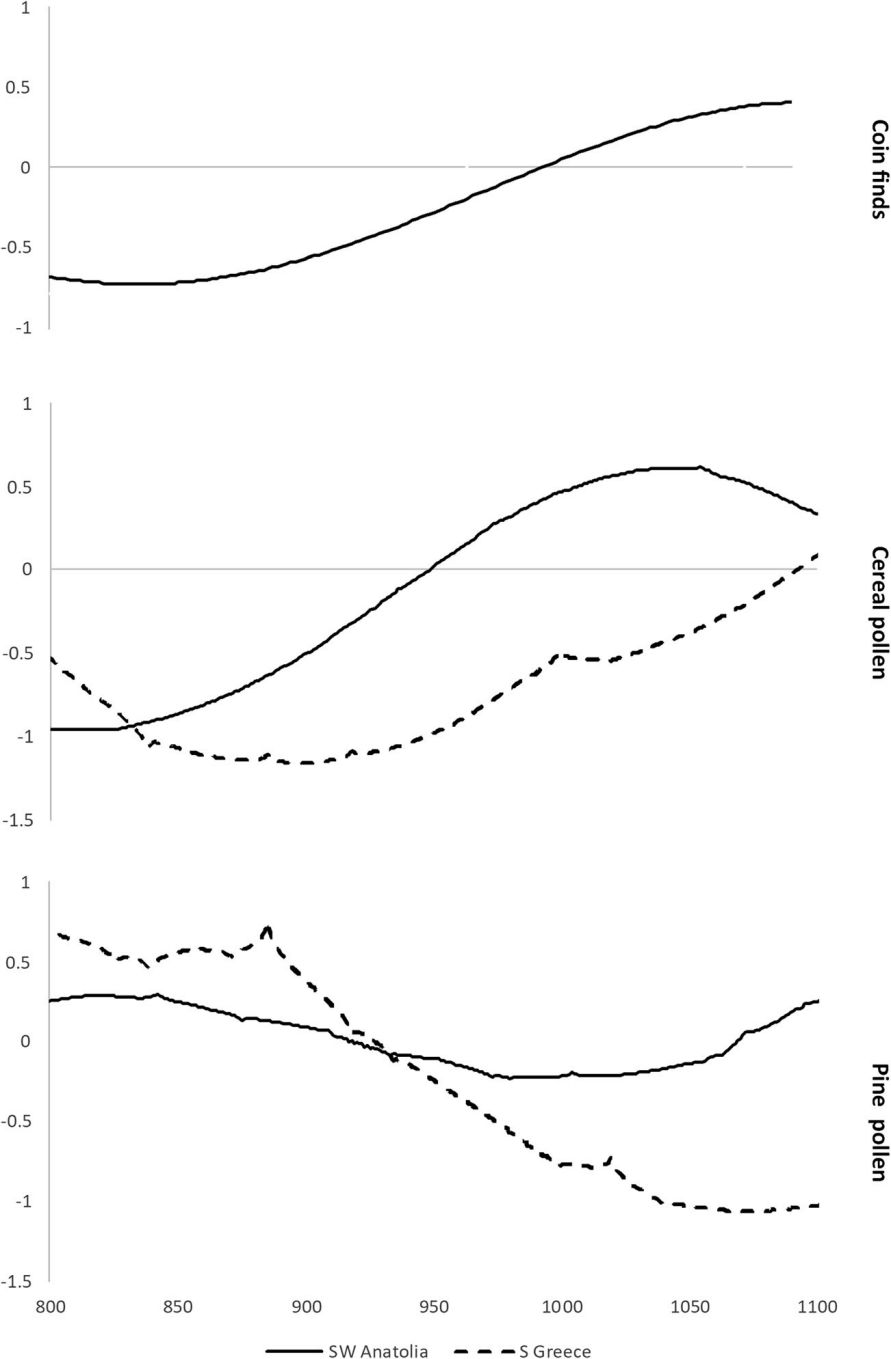
Archaeological and palaeoenvironmental proxies showing that the economic expansion in the Byzantine Empire began already in the 10th c. CE, prior to the “Great Famine” of 927 CE (original values were standardized for the period of 300-1500 CE). Coin finds: Harvey 1989, 86-89; Morrisson 2002 (average of standardized values from individual sites, showing polynomial trend line); pollen data: Izdebski et al. 2015. Pollen and coin find sites are shown on Fig. 1

The apparent discrepancy between the written records and the palaeoclimate data is worth further investigation. First, there is no reason to doubt that a famine occurred in some Byzantine lands in 928 CE. Second, contemporaries seem to have related it to some kind of unusual winter conditions. If we are to take the reports of the written sources at face value, freezing temperatures or frosts would have continued into the spring months (i.e., until April), shortening the vegetation season and impeding seed germination (winter conditions were crucial for the late spring cereal harvest – *Geoponica*, Beckh 1895: II 14). Given the fact that cereals provided some 30-50% of the calories consumed by the medieval population, this could have led to a subsistence crisis (Kaplan 1992: 25-32; Bourbou *et al*. 2011; Zuckerman 2016). Thus, the climate-related shortage-generating mechanism reported by the sources sounds plausible. However, it is striking that this famine occurred at the beginning of a centuries-long period of economic expansion in the Byzantine Empire (Fig. 4). During such a period, one would expect a society to be resilient enough to buffer environmental stress rather than to experience a major climate-related famine.

The necessary clue to understanding what happened in the Byzantine lands in 928 may be found in the legal sources. In their description of the social crisis that resulted from the harsh winter and the poor harvest, they distinguish between the “powerful” and the “weak;” the latter were selling their land to the former for food or money to save their lives. Closer analysis reveals that the “poor” were those engaged in physical labor, while the “powerful” were the Byzantine military and civil officials paid in cash (usually gold) by the imperial government (Morris 1976). For centuries, the government in Constantinople maintained a hierarchy of offices that were paid in gold by the state to ensure the loyalty of its officials, a highly unusual institution in the medieval period. In fact, during the seventh to the tenth century, the gold payments made by the state to its elites were probably the largest transfer of currency occurring every year in Anatolia and the Balkans, and the most important source of revenue for Byzantine elites (Haldon 2009). As a result, the “powerful” Byzantine officials had constant access to liquid assets they could invest in land or any other type of property. This was indeed a rare situation in an age when the Byzantine economy was primarily not monetized and coin finds are usually associated with the activities of the state and its officials, a situation which only started to change in the decades preceding the great winter of 927/928 (see Fig. 4). It is not surprising, therefore, that a climate-related subsistence crisis – even if the weather conditions were actually not *so* unusual compared to the rest of the tenth century – provided them with an opportunity to quickly exchange money for land (for the central role of the socioeconomic context in creating famines even during major climate-related food shortages, see Slavin 2016).

This development eventually increased elite control over the peasants while also disintegrating village communities in the affected provinces. Since the Byzantine taxation system was based on villages organized as fiscal units with communal tax liability (as described in the *Farmer’s Law*, Medvedev 1984; for a broader discussion, see Morrisson 1991; Oikonomidès 1996; Górecki 2004), the impoverishment of peasant producers, the frequent result of selling their land, eventually began to erode the tax base and the social system itself. Thus the “weak” – or peasants – were not the only ones whose situation deteriorated as a result of the “great famine.” The larger-scale socioeconomic change forced the state to intervene in order to protect its resources and attempt to stop the processes of transformation that were amplified by the long winter and the subsistence crisis. Emperor Romanos Lekapenos (920-944) issued a law in 934, limiting the right of the powerful to buy peasants’ land. His goal was first of all to stabilise land ownership patterns and associated tax structures rather than to help those who had recently suffered from the famine. The imperial response also aimed at improving the material situation of the peasantry, but by 934 it was too late for further relief measures to revert the social transformation that had been taking place (Kaplan 1992, p. 421-426). In other words, the emperor’s goal with this particular law was to stop the ongoing social change rather than react directly to the immediate consequences of the winter of 927-928 (which he probably did, but it did not reverse the trend toward the social change either - Wahlgren 2006: 330).

A comparison of the palaeoclimate, pollen, archaeological, and written evidence makes clear that the climate anomaly of 927-28 did not cause substantial social change but rather the long winter was later connected with what the state and the peasants perceived as a socioeconomic crisis. The long winter was thus used as a way of understanding the reasons for the social transformation as it accelerated this process and brought it to contemporaries’ attention. The environmental stressor – even if in physical terms it was not *the* harshest winter of the tenth century – impinged upon the complex web of crop ecologies, social relations, and the state’s interests. Thus, it added new momentum to the extant social dynamic – that of office-holding elites accumulating wealth that allowed them to become an increasingly powerful social group within contemporary Byzantine society.

From a broad perspective, then, Byzantine society proved resilient, surviving the crisis caused by the long winter of 927-28. When seen from the point of view of specific social groups, however, the price for this resilience was a significant shift in the balance of socioeconomic relations. The winter of 927-28 offered an opportunity for the more affluent to take advantage of peasants whose livelihoods depended on ecological niches that were not capable of withstanding the effects of a prolonged cold spell. Thanks to the buffers of their existing estates, their accumulated gold, and their local connections such elite groups could exploit local subsistence crises across the Byzantine provinces in order to improve their situation with regard to both the producing population and the state itself. Moreover, even though there is no concrete evidence, we could expect the same dynamic to take place for other, more local and less extreme environmental (and social) stressors that occurred in the context of the social dynamic of tenth-century society. In this way, environmental stressors and the crises they provoked stimulated social evolution in the Byzantine world.

### The Sixteenth Century: Prolonged Drought and Economic Crisis in the Ottoman Empire

Developments in the Ottoman Empire demonstrate how large-scale state intervention in local settlement and land use could aggravate rather than buffer environmental stress and amplify the scale of social transformation it occasioned. During the late sixteenth through early seventeenth centuries the empire experienced a major crisis triggered by multiple environmental and human stressors, followed by a protracted and intermittent recovery in terms of population, agricultural production, political stability, and military power (as described in more depth in White 2011). This period of Ottoman history provides a well-documented illustration of the burden of resilience in a pre-industrial Eastern Mediterranean society. Although the state and Ottoman dynasty endured, their survival necessitated the abandonment of pre-crisis settlement patterns, provisioning systems, and fiscal arrangements. The burden of this transformation fell principally on the Anatolian *reaya* (peasantry).

Until the crisis, the Ottoman Empire sustained its political and economic resilience throughout rapid territorial expansion. From modest beginnings in northwest Anatolia ca. 1300, Ottoman rulers conquered territory on three continents covering all or part of 30 present-day countries. The empire drew on administratively and geographically diverse sources of tribute, taxation, and requisitions. It adapted pre-Ottoman traditions and developed new systems to mobilize crucial resources from distant locations to provision its cities and military and to balance regions of surplus and deficit. These provisioning systems included food (grains, rice, sheep), labour (human and animal), and strategic materials (timber, gunpowder, alum etc.). The security provided by Ottoman soldiers as well as legal and tax provisions encouraged the expansion of agriculture and the containment of mobile pastoralism. When tested by a series of local droughts, shortages, and famines during the 1560s-1580s, Ottoman officials were able to contain the damage by shifting tax burdens from the affected areas, ordering fixed-price sales of grain from other provinces and in some cases arranging direct shipments from local or imperial granaries (Ágoston 2005; Mikhail 2011).

At the same time, the empire’s growth generated vulnerabilities at both the household and imperial scales. Cadastral surveys and poll tax records (although imperfect sources of data) provide strong indications of rapid population growth. The number of individuals and rural households with little or no land rose much faster than the population as a whole. The overall output of grains and livestock grew, but agriculture faced diminishing marginal returns, shrinking per capita production and therefore limited surplus for imperial provisioning. At the imperial level, the growth of the capital (Istanbul), major cities, army, and navy generated larger demands for resources. To meet the needs of major military campaigns, the empire depended on extraordinary taxation and requisitions from the core provinces in Anatolia and the southern Balkans (Cook 1972; Faroqhi 1984).

**Fig. 5:**
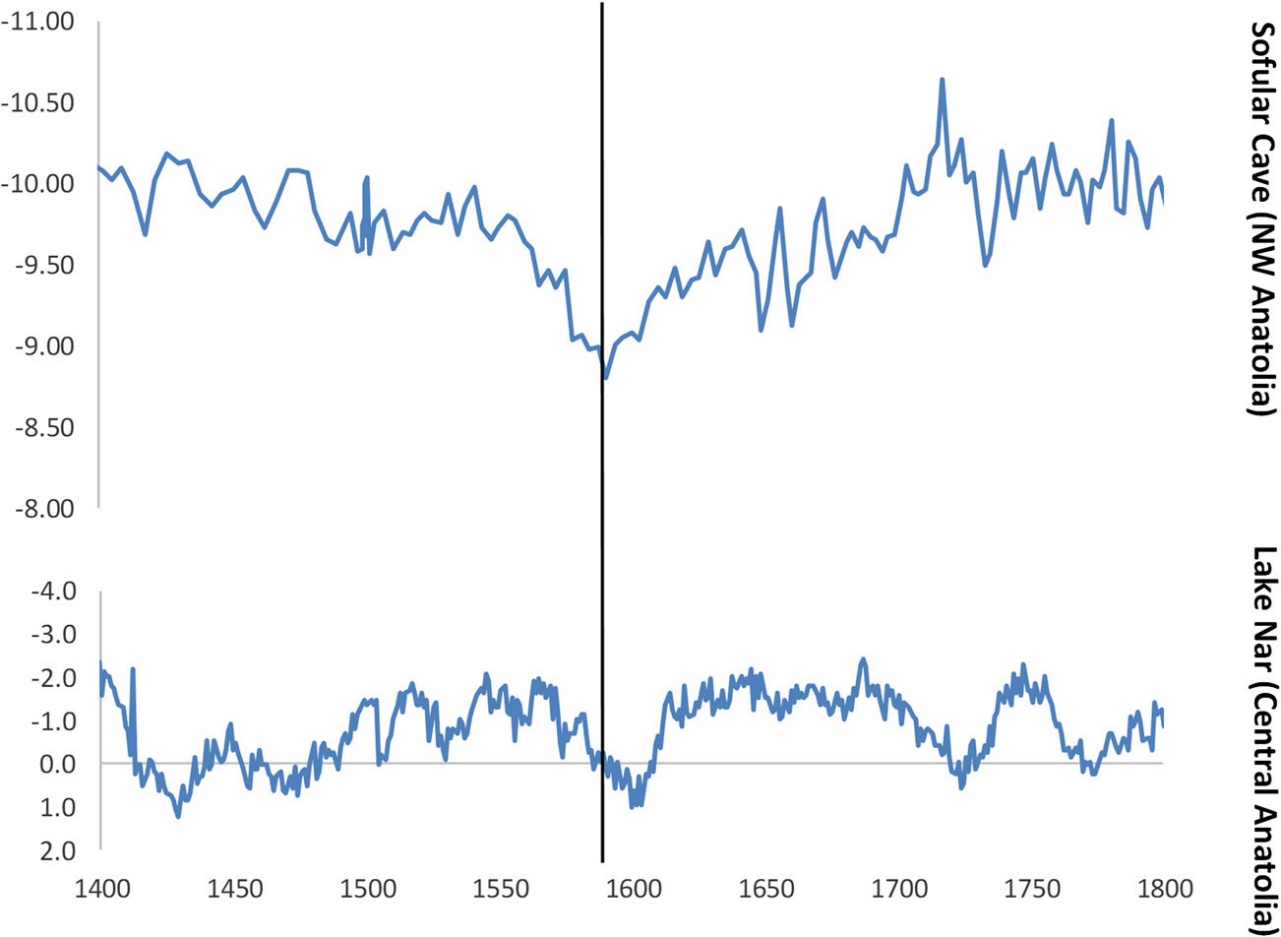
Hydro-climate proxies for Ottoman Anatolia: δ13C values for the Sofular Cave and δ18O for Lake Nar. The values are reversed so that the higher values represent wetter conditions. The black line marks the year AD 1591. Sofular Cave data: Göktürk et al. 2011 (with the revised STALAGE age-depth model). Lake Nar: Jones et al. 2006

Expansion thus buffered Ottoman systems of resource, labour, and military mobilization from small impacts but exposed them to a growing risk of systemic breakdown in the face of multiple, larger shocks. This situation helps explain the scale of crisis in the empire during the 1590s-1600s. During 1591-96, central Anatolia experienced one of its longest and deepest droughts of the past millennium, as described by contemporary sources and confirmed by tree-ring and lake sediment studies (Fig. 5) (Touchan *et al*. 2005; Roberts *et al*. 2012). Drought during the spring growing season appears to have been especially damaging to the grain crop in central and western Anatolia during the middle years of the decade. Food prices more than doubled and some primary accounts suggest food was often unavailable. During and after those years of drought, there are anecdotal descriptions of extraordinarily cold winters, a phenomenon possibly linked to large tropical volcanic eruptions, including Nevado del Ruiz (1595) and Huaynaputina (1600) (Sigl *et al*. 2015; Xoplaki *et al*. 2018). The combination of drought and cold likely contributed to the outbreak of a major epizootic disease, which affected sheep and cattle across Anatolia, the Crimea, and the Balkans, eventually passing through Hungary into Central Europe. The death of livestock deprived Ottoman peasants of a major source of wealth and subsistence, and deprived Ottoman armies of a key source of protein (White 2017).

The Ottoman Empire might have been able to contain the crisis but for the distractions and demands of military campaigns during the so-called Long War (1593-1607) with the Habsburg Empire. Rather than reducing taxation or providing relief supplies, as during previous droughts and famines, Ottoman rulers actually increased requisitions from the worst hit Balkan and Anatolian provinces, escalating shortages and famines. Imperial demands for sheep from Karaman province (the region around today’s Konya) appear to have been the proximate cause for a major rural uprising, the Celâlî Rebellion (1596-1610). Positive feedback among famine, violence, population displacement, and contagious disease generated a significant mortality crisis in parts of the empire. Many households were displaced from rural to urban areas, which faced higher levels of endemic and epidemic disease and mortality. Pastoralists reoccupied much farmland abandoned during this mortality peak, which precluded its immediate return to agriculture. Although exact figures are impossible to come by, household counts in certain tax records from the 1620s-30s suggest losses of half or more in many parts of Anatolia since the 1580s (Özel 2016).

These decades of crisis witnessed what has been termed a “transformation” of political and fiscal structures, or even the beginning of a “second Ottoman Empire,” involving the modification or abandonment of some pre-crisis provisioning systems, systems of revenue-raising based on land holdings, and military service in return for assignments of local revenues. In their place, the empire resorted to cash taxes levied collectively on groups of rural households as well as the sale of short-term tax farms and the use of irregular mercenary armies (*sekban*). Although these measures diminished the personal authority of Ottoman sultans and entailed significant decentralization of power, they enabled the imperial government to co-opt potentially rebellious elites and to pay soldiers frequently enough that the state and dynasty survived the extraordinary turmoil of the early seventeenth century and eventually developed more stable political and fiscal institutions (Darling 1996; Tezcan 2010).

This political survival, however, imposed significant burdens on Anatolian peasants. The state could no longer grant tax relief or implement sales or distributions of grain to provinces facing poor harvests and shortages during the frequent adverse climate conditions during the seventeenth century. The immediate demands of short-term tax farmers clashed with the need to restore stability and investment in agriculture. State officials were no longer in a position to ensure the tax exemptions and security from banditry that had previously supported the expansion of rural settlement. Communities facing violence and population loss, unable to pay taxes levied on them collectively, often fled to towns and cities, where they faced periodic persecution and expulsion (White 2011). The costs of imperial resilience thus fell on those least able to bear it.

## Conclusions

These case studies reveal the implications, or social burden, of resilience in highly complex pre-modern societies. In all three cases, the state as the central institution demonstrated resilience to combined socio-environmental stressors over a period of a few years to several decades. The scholarly focus on the state in these cases often derives from the simple fact that more surviving evidence covers this level of a given social system. Keeping the focus on the state, however, occludes the parallel processes taking place at other levels of a society.

The case study of the sixth century Roman Empire demonstrates the difficulties a centralized decision-making apparatus such as a government encounters when it attempts to balance its interests (sustainable survival) with those of the institutions and sectors under its influence (e.g., individual cities, industries, or even elite constituencies). The tension between these sets of interests and the power relations embedded in and between the social system and its sub-systems shapes their response to stress. This set of responses, in turn, becomes the reality within which individuals operate and make their own decisions.

The case of Byzantium emphasizes the long-term unforeseen implications of environmental stress and the extent to which the social context determines the actual socio-economic impact of even a relatively modest stressor. It demonstrates that the societal effects of such stressors can develop slowly and over time while accelerating existing trends or exploiting existing vulnerabilities in society. The long winter and subsistence crisis strained weaker social groups in Byzantine society; stronger, wealthier groups of elites – with connections to government structures – were not only better buffered to stave off the crisis, but also exploited it to their advantage. This in turn exacerbated the crisis for the weaker groups and undermined the state’s potential capability to mitigate its effects, triggering a major famine.

The Ottoman Empire illustrates how political resilience could impose differential social and economic costs. For the state to endure the crisis of the late sixteenth to early seventeenth centuries it had to resort to short-term expedients that shifted burdens onto rural populations and undercut elements of provincial provisioning and security. The effects of the state’s approach cascaded into longer-term changes, with large numbers of the impoverished rural communities moving into urban areas, increasing social unrest and decreasing the empire’s tax base.

The cases all demonstrate that socio-ecological resilience incurs differential burdens across the levels of a social system, and can further impose collateral costs at the same hierarchical level (whereas the classical panarchy model tends to emphasize effects between the different hierarchical levels of the same system, see, for example, Holling 2001; Gunderson and Holling 2002). Certain institutions, social groups and individuals pay more – a cost that can reach up to and including their lives. In parallel, others can improve their position. As such, socio-environmental stress can lay bare the configurations of power within a social system as well as its working and failing. Moreover, our case studies demonstrate that the impacts of environmental stressors, and of the disasters associated with their occurrence are themselves socially constructed. They are modified by social circumstances and are articulated through the networks of political institutions, social actors, economic relations and cultural phenomena in which they are embedded (Janku *et al*. 2012; Schenk 2017).

Despite their power and potential to dominate the exploitation of available resources, pre-modern states did not necessarily fare best, as illustrated by the case of tenth century Byzantium. Vacuums created by the weakened state’s absence, such as the former farmland in the sixteenth-century Ottoman Empire, quickly can be taken over by more adaptive non-elite groups, as was the case with the nomadic pastoralists who moved in to replace the cultivators who had abandoned their lands. By the same token, however, neither do all elites fare better than all non-elites. The elites who lived in sixth-century Apamea and Berytus lost, while villagers who moved into abandoned urban houses, or the artisans of the silk industry in Constantinople, probably gained. A major difference between elites and non-elites, however, is in the greater buffering capacity of the former. While the elites of Apamea and perhaps Berytus could relocate elsewhere, albeit with a cost to their well-being and personal finances, the risk faced by non-elites such as those employed in Berytus’ silk industry was substantially higher.

These historical case studies provide important insights into the discussion within current research on social-environmental resilience as the “gold standard” for future policy-making, in the context of growing environmental concerns (e.g., global climate change; for problems associated with this approach, see Cote and Nightingale 2012; Olsson *et al*. 2015; Olsson 2017). They demonstrate the importance of being aware of implicit value judgements as well as the interests of all social groups involved, even those which are normally not involved as stakeholders in decision-making processes. By contextualizing resilience, our work touches upon the notion of environmental justice, namely the fair treatment of all people with respect to the benefits they can draw from the environment, and the risks from it to which they are exposed. In this context, we can suggest that the deep roots of some of the social challenges of the twenty-first century, including poverty, can be traced back to mechanisms that are much older than our contemporary global order (Fitzpatrick 2014). Moreover, as our case studies demonstrate, these challenges are integral to the structure of a social-economic system itself and are not in themselves caused by climate change or any other environmental stressor. This should assist in recognising both the real scale of the problem and in encouraging more comprehensive approaches to its solutions.

There can be no doubt of the value of studying the historical past for resilience theory in light of the countless surviving preindustrial case studies that demonstrate social responses to environmental stressors (on integrating history and palaeoscience, see Haldon *et al*. 2014; Izdebski *et al*. 2016; Haldon *et al.* 2018). These case studies offer observers the opportunity to trace the results of socio-environmental interactions retrospectively over different chronological time frames. The wide spatial perspective offered by the historical examples presented here permits consideration of the interests of and outcomes for the multiple subgroups that are the subject of our investigation. By the same token, historical research also reveals the importance of taking into account the interests, perceptions, and beliefs of all those involved within a system when considering questions of resilience (Haldon and Rosen, this issue).
